# Clear Cell Renal Cell Carcinoma Metastasized to the Ampulla of Vater 16 Years after Nephrectomy—A Rare Case

**DOI:** 10.3390/diagnostics12030571

**Published:** 2022-02-23

**Authors:** Jun Lu, Weijiang Zhou, Xuyong Wei, Kai Wang, Lixin Zhou, Xiao Xu

**Affiliations:** 1 Department of Hepatobiliary and Pancreatic Surgery, Affiliated Hangzhou First People’s Hospital, Zhejiang University School of Medicine, Hangzhou 310006, China; 13788989913@163.com (J.L.); zhouweijiang2009@163.com (W.Z.); 1315009@zju.edu.cn (X.W.); kaiw3@zju.edu.cn (K.W.); 2 Zhejiang Provincial Key Laboratory of Integrated Oncology and Intelligent Medicine, Hangzhou 310006, China

**Keywords:** renal cell carcinoma, periampullary metastasis, pancreaticoduodenectomy, interesting images

## Abstract

Although clear cell renal cell carcinoma (ccRCC) is easy to diagnose early and most can be radically resected, nearly one-third of patients still experience metastases after radical nephrectomy. The most common distant metastases sites of ccRCC are lung, bone and liver. However, periampullary metastasis of ccRCC is very rare and easy to misdiagnose. A 59-year-old male patient was hospitalized for recurrent hematochezia. He had a history of nephrectomy 16 years ago due to ccRCC. Enhanced upper abdominal computed tomography (CT) suggested a mass in the ampulla of vater, and active hemorrhage of duodenal papilla was observed by endoscopy. He underwent an emergency pancreaticoduodenectomy because endoscopic hemostasis and transcatheter arterial embolization (TAE) both failed. Intraoperatively, we found that the tumor located in the ampulla and invaded the pancreatic tissue. The operation was successful, with no postoperative complications. Postoperative pathology suggested metastatic ccRCC.

**Figure 1:**
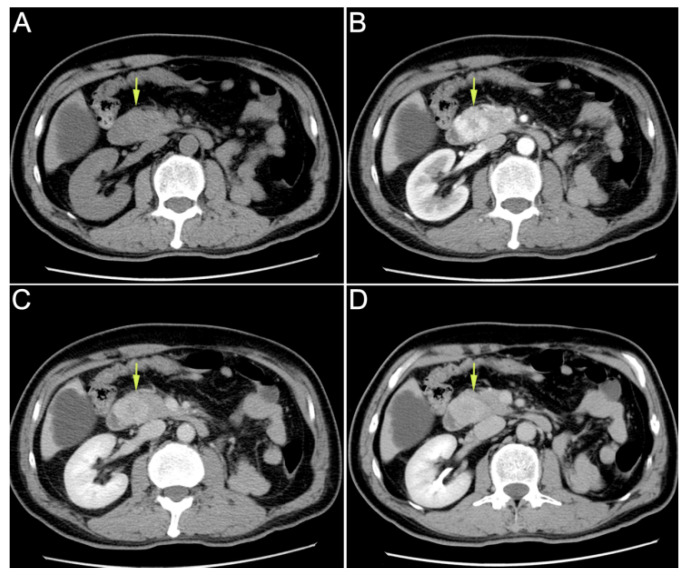
Enhanced upper abdominal computed tomography (CT) performed in the local hospital indicated ampullary mass. (**A**): plain scan phase; (**B**): arterial phase; (**C**): venous phase; (**D**): delayed phase. A 59-year-old male patient was hospitalized for “recurrent hematochezia for 8 days”. Enhanced upper abdominal computed tomography (CT), performed in the local hospital, indicated ampullary mass. On CT, the tumor showed uneven enhancement in the arterial phase, but the enhancement significantly weakened in the venous phase and delayed phase. Therefore, this imaging feature was significantly different from that in the primary ampullary tumor or pancreatic tumor. Duodenoscopy revealed duodenal papilla hemorrhage. Urinalysis showed no hematuria. After conservative treatment, the hemorrhage was not resolved. Then, the patient was transferred to our hospital for further treatment. He had received radical nephrectomy due to clear cell renal cell carcinoma (ccRCC) of the left kidney 16 years ago. The CT scan mentioned above revealed that his right kidney was normal. On admission, his laboratory tests results were as follows: leukocyte count of 3.9 × 10^9^/L; neutrophilic granulocytes percentage of 75%; C-reactive protein of 1.6 mg/L; hemoglobin of 102 g/L; total bilirubin concentration of 12.6 μmol/L; alanine aminotransferase of 16 U/L; aspartate aminotransferase of 19 U/L.

**Figure 2:**
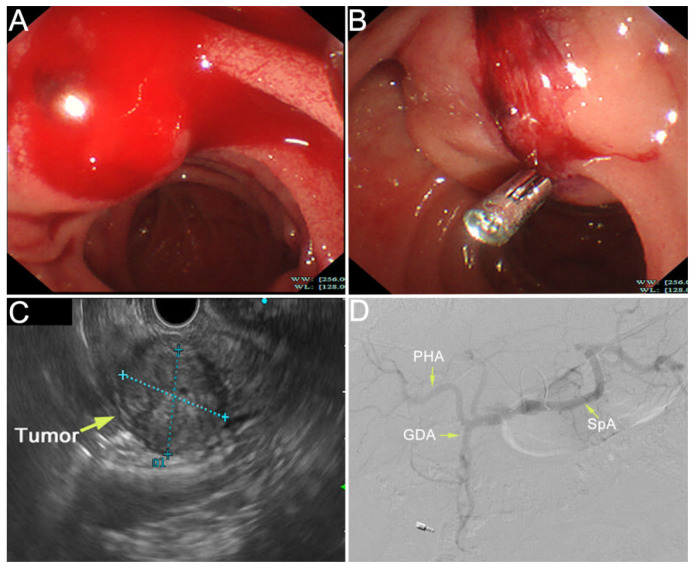
The duodenal papilla was plump with pulsatile bleeding by emergency duodenoscopy (**A**). The ampullary mass was observed by endoscopic ultrasonography, with a size of 22.7 × 28.2 mm (**C**). We diagnosed that the tumor had invaded the artery and the bleeding site was indistinct due to the persistent bleeding. Hemostasis was performed by using an endoscopic metal clip and the hemorrhage was partially relieved (**B**). However, due to the fragility of the tumor, complete hemostasis was difficult. Then, we tried to perform TAE in hemostasia. However, there were no obvious signs of contrast extravasation (**D**). After a Multi-Disciplinary Treatment (MDT), we decided to perform emergency pancreaticoduodenectomy, which was recommended by most experts on MDT. Intraoperatively, it was found that the tumor was located in the ampulla of vater and had invaded the pancreatic tissue. Modified Child anastomosis was performed after radical pancreaticoduodenectomy: gastrojejunostomy (Braun’s anastomosis), pancreaticojejunostomy (anastomosis of pancreatic duct with jejunal mucosa through a tent duct), and biliojejunostomy (end-to-side). He also underwent fenestration for a cyst in his right liver. The operation went well.

**Figure 3:**
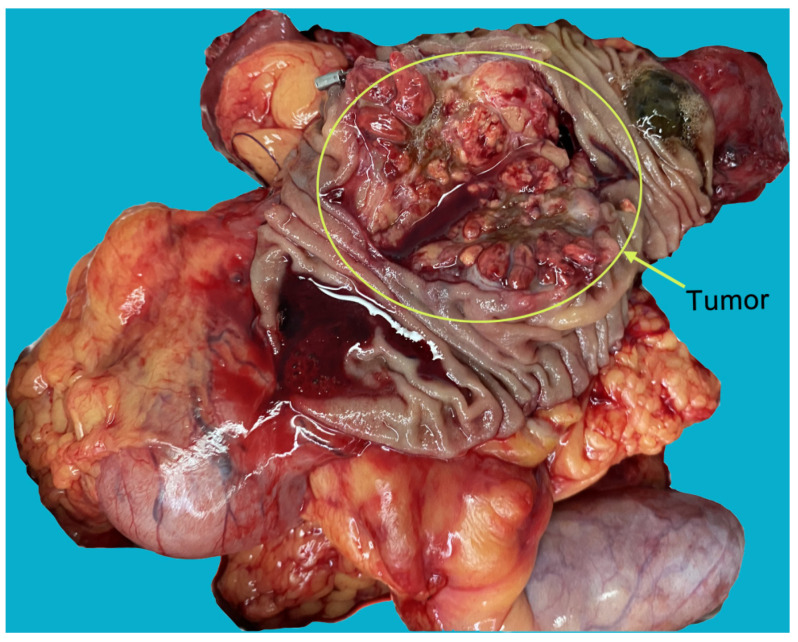
The specimens showed that the tumor was located in the ampulla, and had invaded the pancreatic tissue. The morphology and texture of the tumors were markedly different from primary ampullary and pancreatic tumors.

**Figure 4:**
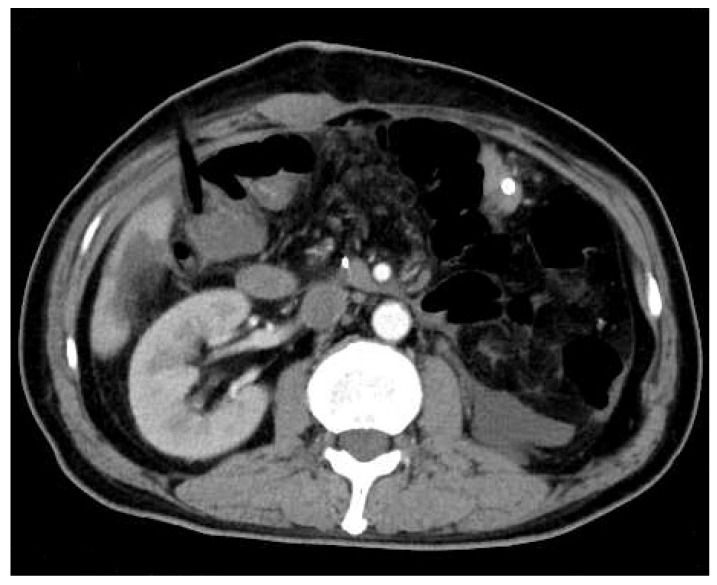
Abdominal enhanced computed tomography (CT) was rechecked on the fourth postoperative day. It showed no obvious effusion or residual lesion in the abdominal cavity.

**Figure 5:**
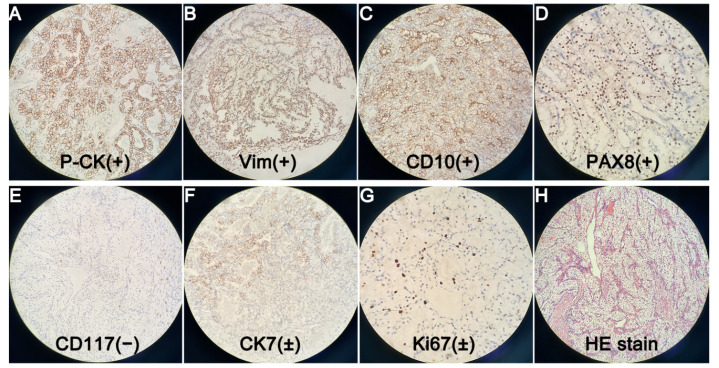
(×200). Postoperative pathology showed metastatic ccRCC, with Fuhrman nuclear grade I. Immunohistochemical staining suggested Pan Cytokeratin (P-CK) (+) (**A**). Vimentin (Vim) (+) (**B**). Cluster of Differentiation 10 (CD10) (+) (**C**). Paired Box Gene 8 (PAX8) (+) (**D**). Cluster of Differentiation 117 (CD117) (−) (**E**). Cytokeratin 7 (CK7) (±) (**F**). Cell Proliferation Antigen Ki67 (Ki67) (±) (**G**). Other markers such as Renal Cell Carcinoma Marker (RCC-Ma), Transcription Factor Binding To IGHM Enhancer 3 (TFE3), Carbohydrate Antigen 199 (CA199) were all negative, but they were not shown in the figure. We also showed a figure of HE staining (**H**). Emergency endoscopic hemostasis and transcatheter arterial embolization (TAE) are both good optional treatments for upper gastrointestinal bleeding [1,2]. However, both of them failed in this case. Therefore, we had to perform emergency pancreaticoduodenectomy. Although complications after emergency pancreatectomy were reported to be much higher than those after elective surgery [3], there were no postoperative complications such as pancreatic fistula, biliary fistula and intestinal fistula. The patient was discharged 16 days after surgery. According to reports, the most common distant metastases sites of ccRCC are lung, bone, liver, etc. [4]. Periampullary metastasis of ccRCC is very rare. To the best of our knowledge, only 19 cases have been reported in the world to date [5,6,7,8]. Good pathological differentiation, isolation or less metastases, heterochronous disease (disease free interval > 2 years), no progression of systemic treatment, low or moderate Fuhrmann grade, and complete resection were associated with good prognosis after local treatment of RCC metastases [9]. Therefore, we estimated that the patient would have a good prognosis. Total resection of metastases can improve overall survival and cancer-specific survival [10]. Therefore, Positron Emission Tomography-Computed Tomography (PET-CT) was necessary for this patient to avoid missing other metastases. Lenvatinib plus pembrolizumab is recommended as the first-line treatment for advanced ccRCC [11]. Therefore, we repeatedly recommended that the patient should return to the hospital one month after surgery for anti-tumor immunotherapy, and have a PET-CT examination to exclude metastases in other organs. However, the patient refused further follow-up and treatment.

## Data Availability

The data presented in this manuscript are available from the corresponding authors upon request.
